# Supplementary material for: High-resolution directed human connectomes and the Consensus Connectome Dynamics
Source: PLoS One. 2019 Apr 16;14(4):e0215473. doi: 10.1371/journal.pone.0215473 (PMC6467387; doi:10.1371/journal.pone.0215473)
Supplement: S1 Table — (PDF) [file pone.0215473.s001.pdf]

Supporting material for the article: B Szalkai, C Kerepesi, B Varga, V Grolmusz: High-Resolution Directed Human Connectomes and the Consensus Connectome Dynamics

The source code of the program computing the direction of the connectome edges:

```
using Satsuma;
using Satsuma.IO.GraphML;
using System;
using System.Collections.Generic;
using System.IO;
using System.Linq;
using System.Text;
using System.Threading.Tasks;
using System.Diagnostics;

namespace BalazsDirection
{
        class Utils
        {
        public static void Swap<T>(ref T x, ref T y)
        {
            T tmp = x;
            x = y;
            y = tmp;
        }
        }

        class Program
        {
        // can be 1 or 2
        const int algorithmVersion = 1;
        static readonly string versionStr = (algorithmVersion == 1 ? "" :
"_"+algorithmVersion);
        const bool doNotDirectInnerEdges = (algorithmVersion == 2);

        static int LogicalOr(int a, int b)
                {
                        if (a != 0)
                                return a;
                        return b;
                }

        class ArcData
        {
            public string Name; // node1~node2, where node1 precedes node2
lexicographically
            public List<int> Directions;

            public int Direction; // -1: undef, 0: forward, 1: backward
            public int ContainingDivisions() { return Directions.Count; } // number
of divisions containing
this edge
            public int MajoritySize; // number of divisions with majority verdict
        }

        Dictionary<string, ArcData> arcData = new Dictionary<string, ArcData>();
        Dictionary<string, string> rowForEdge = new Dictionary<string, string>();
        Dictionary<string, double[]> nodePosByName = new Dictionary<string,
double[]>();

        // start: index of start graphml in list
        // end: index of end graphml in list
        void Process(List<string> graphmls, int start, int end)
                {
            string nodesCsv = "graph_nodes" + versionStr + "_" + start + "_" + end
+ ".csv";
            string arcsCsv = "graph_arcs" + versionStr + "_" + start + "_" + end +
```

```
".csv";
            string arcFreqFileName = "arcfreq" + versionStr + "_" + start + "_" +
end + ".csv";
            string outGraphml = "directed_graph" + versionStr + "_" + start + "_" +
end + ".graphml";
            if (File.Exists(nodesCsv) && File.Exists(arcsCsv))
            {
                // load edge direction data
                foreach (var line in File.ReadLines(arcsCsv))
                {
                    int semicolon = line.IndexOf(';');
                    if (semicolon < 0)
                        continue;
                    string edgeName = line.Substring(0, semicolon);
                    string row = line.Substring(semicolon+1);
                    if (!rowForEdge.ContainsKey(edgeName))
                        rowForEdge[edgeName] = "";
                    rowForEdge[edgeName] += row;

                    string[] t = line.Split(';');
                    int arcDir = int.Parse(t[t.Length - 2]);

                    if (!arcData.ContainsKey(edgeName))
                    {
                        arcData[edgeName] = new ArcData();
                        arcData[edgeName].Directions = new List<int>();
                    }
                    arcData[edgeName].Name = edgeName;
                    arcData[edgeName].Directions.Add(arcDir);
                }
                return;
            }

            // load the graphs and create the sum graph
            CustomGraph sumGraph = new CustomGraph();
                        Dictionary<Node, string> nodeName = new Dictionary<Node,
string>();
//            Dictionary<Node, double[]> nodePos = new Dictionary<Node,
double[]>();
            Dictionary<string, Node> nodeByName = new Dictionary<string, Node>();
                        Dictionary<Arc, int> arcFrequency = new Dictionary<Arc,
int>();
            int maxArcFrequency = 0;
                        for (int i = start; i < end; ++i)
                        {
                                GraphMLFormat f = new GraphMLFormat();
                                Console.WriteLine(graphmls[i]);
                                try
                                {
                                        f.Load(graphmls[i]);
                                }
                                catch
                                {
                                        continue;
                                }
                                IGraph g = f.Graph;
                                var pName =
(StandardProperty<string>)f.Properties.Where(p => p.Name ==
"dn_name").First();
                var px = (StandardProperty<double>)f.Properties.Where(p => p.Name
== "dn_position_x").First
();
                var py = (StandardProperty<double>)f.Properties.Where(p => p.Name
== "dn_position_y").First
();
                var pz = (StandardProperty<double>)f.Properties.Where(p => p.Name
== "dn_position_z").First
();
                if (sumGraph.NodeCount() == 0)
                                        foreach (var n in g.Nodes())
                                        {
                                                Node n2 = sumGraph.AddNode();
```

```
                                        nodeName[n2] = pName[n];
                                        nodeByName[nodeName[n2]] = n2;
                        nodePosByName[nodeName[n2]] = new double[] { px[n], py[n],
pz[n] };
                    }
                                foreach (var a in g.Arcs())
                                {
                                        Node u = g.U(a);
                                        Node v = g.V(a);
                                        Node u2 = nodeByName[pName[u]];
                                        Node v2 = nodeByName[pName[v]];
                                        Arc aInSumGraph = sumGraph.Arcs(u2,
v2).FirstOrDefault();
                                        if (aInSumGraph == Arc.Invalid)
                                        {
                                                aInSumGraph = sumGraph.AddArc(u2,
v2, Directedness.Undirected);
                                                arcFrequency[aInSumGraph] = 0;
                                        }
                                        arcFrequency[aInSumGraph]++;
                    if (arcFrequency[aInSumGraph] > maxArcFrequency)
                        maxArcFrequency = arcFrequency[aInSumGraph];
                }
                        }
            using (StreamWriter sw = new StreamWriter(arcFreqFileName))
            {
                foreach (var kv in arcFrequency)
                {
                    var arc = kv.Key;
                    string uName = nodeName[sumGraph.U(arc)];
                    string vName = nodeName[sumGraph.V(arc)];
                    if (uName.CompareTo(vName) > 0)
                        Utils.Swap(ref uName, ref vName);
                    var arcName = uName + "~" + vName;
                    sw.WriteLine(arcName + ";" + kv.Value);
                }
            }
                        // determine the step when a point became connected
                        Dictionary<Node,int> nodeLevel = new
Dictionary<Node,int>();
                        int maxLevel = 0;
                        foreach (var n in sumGraph.Nodes())
                        {
                                nodeLevel[n] = sumGraph.Arcs(n).Select(a =>
arcFrequency[a]).MaxOr(0);
                                maxLevel = Math.Max(maxLevel, nodeLevel[n]);
                        }
                        Dictionary<Node, int> nodeDist = new Dictionary<Node,
int>(); // distance from next
level
            Subgraph eddigiÉlek = new Subgraph(sumGraph);
            eddigiÉlek.EnableAllNodes(true);
                        foreach (var n in sumGraph.Nodes())
                        {
                foreach (var a in sumGraph.Arcs())
                    eddigiÉlek.Enable(a, arcFrequency[a] >= nodeLevel[n]);
                Bfs bfs = new Bfs(eddigiÉlek);
                                bfs.AddSource(n);
                                Node parent = bfs.RunUntilReached(x => nodeLevel[x]
> nodeLevel[n]);
                                if (parent == Node.Invalid)
                                        nodeDist[n] = int.MaxValue;
                                else
                                        nodeDist[n] = bfs.GetLevel(parent);
                        }
                        List<Node> nodes = sumGraph.Nodes().ToList();
                        nodes.Sort((a, b) => LogicalOr(nodeLevel[b]-nodeLevel[a],
nodeDist[a]-nodeDist[b]));
            using (StreamWriter sw = new StreamWriter(nodesCsv))
                        {
                                foreach (var n in nodes)
                                {
```

```
                                        sw.WriteLine(nodeName[n] + ";" +
nodeLevel[n] + ";" + nodeDist[n]);
                                }
                        }
            // direct the edges
            CustomGraph irányítás = new CustomGraph();
            Dictionary<Node, Node> irányítottCsúcs = new Dictionary<Node, Node>();
            foreach (var n in sumGraph.Nodes())
                irányítottCsúcs[n] = irányítás.AddNode();

            HashSet<Arc> mustBeUndirected = new HashSet<Arc>();
            if (doNotDirectInnerEdges)
            {
                // don't direct those edges which were created between two points
                // already in the same component

                // first, order the edges by frequency increasing
                // (we will use them by freq decreasing actually)
                List<Arc> arcsByFreq = sumGraph.Arcs().ToList();
                arcsByFreq.SortByKey(a => arcFrequency[a]);
                DisjointSet<Node> connectedComponents = new DisjointSet<Node>();
                int endOfArcRange = arcsByFreq.Count;
                for (int freq = maxArcFrequency; freq >= 1; --freq)
                {
                    int startOfArcRange = endOfArcRange;
                    while (startOfArcRange > 0 &&
arcFrequency[arcsByFreq[startOfArcRange - 1]] >= freq)
                        --startOfArcRange;
                    for (int i = startOfArcRange; i < endOfArcRange; ++i)
                    {
                        Arc a = arcsByFreq[i];
                        Node u = sumGraph.U(a);
                        Node v = sumGraph.V(a);
                        if (connectedComponents.WhereIs(u) ==
connectedComponents.WhereIs(v))
                            // arc appears within an already connected component
                            mustBeUndirected.Add(a);
                    }
                    for (int i = startOfArcRange; i < endOfArcRange; ++i)
                    {
                        Arc a = arcsByFreq[i];
                        Node u = sumGraph.U(a);
                        Node v = sumGraph.V(a);
                        connectedComponents.Union(connectedComponents.WhereIs(u),
connectedComponents.WhereIs(v));
                    }
                    endOfArcRange = startOfArcRange;
                }
                Console.WriteLine(mustBeUndirected.Count + " arcs will become
undirected by the new
method");
            }

            using (StreamWriter sw = new StreamWriter(arcsCsv))
                        {
                                foreach (var a in sumGraph.Arcs())
                                {
                                        Node u = sumGraph.U(a);
                                        Node v = sumGraph.V(a);
                                        if (nodeName[u].CompareTo(nodeName[v]) > 0)
                                                Utils.Swap(ref u, ref v);

                                        Node arcTo = Node.Invalid;
                    Node arcFrom = Node.Invalid;
                                        int arcDir = -1; // undirected
                    if (!mustBeUndirected.Contains(a))
                    {
                        int compareResult = LogicalOr(nodeLevel[v] - nodeLevel[u],
nodeDist[u] - nodeDist
[v]);
                        if (compareResult < 0)
                        {
```

```
                            arcFrom = v;
                            arcTo = u;
                            arcDir = 1;
                        }
                        else if (compareResult > 0)
                        {
                            arcFrom = u;
                            arcTo = v;
                            arcDir = 0;
                        }
                    }

                    Debug.Assert((arcTo == Node.Invalid) == (arcDir == -1));
                    if (arcTo == Node.Invalid)
                        irányítás.AddArc(irányítottCsúcs[u], irányítottCsúcs[v],
Directedness.Undirected);
                    else
                        irányítás.AddArc(irányítottCsúcs[arcFrom],
irányítottCsúcs[arcTo],
Directedness.Directed);

                    string edgeName = nodeName[u] + "~" + nodeName[v];
                                        string row = start+";"+end+";"+nodeName[u]
+ ";" + nodeName[v] + ";" +
arcFrequency[a] + ";" + arcDir + ";";
                                        if (!rowForEdge.ContainsKey(edgeName))
                                                rowForEdge[edgeName] = "";
                                        rowForEdge[edgeName] += row;
                                        sw.WriteLine(edgeName + ";" + row);

                    if (!arcData.ContainsKey(edgeName))
                    {
                        arcData[edgeName] = new ArcData();
                        arcData[edgeName].Directions = new List<int>();
                    }
                    arcData[edgeName].Name = edgeName;
                    arcData[edgeName].Directions.Add(arcDir);
                }
            }

            // output the directed graph as a graphml
            GraphMLFormat graphmlOut = new GraphMLFormat();
            graphmlOut.Graph = irányítás;
            var irányított_nodeName = new StandardProperty<string>();
            irányított_nodeName.Domain = PropertyDomain.Node;
            irányított_nodeName.Name = "dn_name";
            foreach (Node n in nodeName.Keys)
                irányított_nodeName.Values[irányítottCsúcs[n]] = nodeName[n];
            graphmlOut.Properties.Add(irányított_nodeName);
            for (int i = 0; i < 3; ++i)
            {
                var irányított_nodePos = new StandardProperty<double>();
                irányított_nodePos.Domain = PropertyDomain.Node;
                irányított_nodePos.Name = "dn_position_" + "xyz"[i];
                foreach (string nn in nodePosByName.Keys)
                    irányított_nodePos.Values[irányítottCsúcs[nodeByName[nn]]] =
nodePosByName[nn][i];
                graphmlOut.Properties.Add(irányított_nodePos);
            }
            graphmlOut.Save(outGraphml);
                }

        int GetVerdict(int undefCount, int forwardCount, int backwardCount)
        {
            int totalCount = undefCount + forwardCount + backwardCount;
            int verdict = -1;
            if (forwardCount >= 2 && totalCount - forwardCount <= 1)
                verdict = 0;
            else if (backwardCount >= 2 && totalCount - backwardCount <= 1)
                verdict = 1;
            return verdict;
        }
```

```
        static void CreateDirectedVersion(string fngraphml,
Dictionary<string,ArcData> arcData)
        {
            string fnout = fngraphml.ReplaceAtEnd(".graphml", "_directed.graphml");
            if (File.Exists(fnout))
            {
                Console.WriteLine(fnout + " already exists");
                return;
            }
            Console.WriteLine("Directed version of " + fngraphml);
            GraphMLFormat f = new GraphMLFormat();
            try
            {
                f.Load(fngraphml);
            }
            catch (Exception e)
            {
                Console.WriteLine("Could not load!");
                Console.WriteLine(e);
                return;
            }
            CustomGraph g = (CustomGraph)f.Graph;

            var pName = (StandardProperty<string>)f.Properties.Where(p => p.Name ==
"dn_name").First();
            var number_of_fibers = f.Properties.Where(p => p.Name ==
"number_of_fibers").First() as
StandardProperty<int>;
            var FA_mean = f.Properties.Where(p => p.Name == "FA_mean").First() as
StandardProperty<double>;
            var FA_std = f.Properties.Where(p => p.Name == "FA_std").First() as
StandardProperty<double>;
            var fiber_length_mean = f.Properties.Where(p => p.Name ==
"fiber_length_mean").First() as
StandardProperty<double>;
            var fiber_length_std = f.Properties.Where(p => p.Name ==
"fiber_length_std").First() as
StandardProperty<double>;

            var edge_MajoritySize = new StandardProperty<int>();
            edge_MajoritySize.Domain = PropertyDomain.Arc;
            edge_MajoritySize.Name = "dn_majority_size";
            f.Properties.Add(edge_MajoritySize);

            var edge_NumberOfDivisions = new StandardProperty<int>();
            edge_NumberOfDivisions.Domain = PropertyDomain.Arc;
            edge_NumberOfDivisions.Name = "dn_prevalence";
            f.Properties.Add(edge_NumberOfDivisions);

            List<Arc> arcs = g.Arcs().ToList();
            foreach (var a in arcs)
            {
                Node u = g.U(a);
                Node v = g.V(a);
                string uname = pName.Values[u];
                string vname = pName.Values[v];
                if (uname.CompareTo(vname) > 0)
                {
                    Utils.Swap(ref u, ref v);
                    Utils.Swap(ref uname, ref vname);
                }

                string edgeName = uname + "~" + vname;
                if (!arcData.ContainsKey(edgeName))
                    continue;

                ArcData ad = arcData[edgeName];

                // redirect the arc
                Arc theArc = a;
                switch (ad.Direction)
```

```
                {
                    case 0:
                        g.DeleteArc(a);
                        theArc = g.AddArc(u, v, Directedness.Directed);
                        break;

                    case 1:
                        g.DeleteArc(a);
                        theArc = g.AddArc(v, u, Directedness.Directed);
                        break;
                }

                edge_MajoritySize.Values[theArc] = ad.MajoritySize;
                edge_NumberOfDivisions.Values[theArc] = ad.ContainingDivisions();

                number_of_fibers.Values[theArc] = number_of_fibers.Values[a];
                FA_mean.Values[theArc] = FA_mean.Values[a];
                FA_std.Values[theArc] = FA_std.Values[a];
                fiber_length_mean.Values[theArc] = fiber_length_mean.Values[a];
                fiber_length_std.Values[theArc] = fiber_length_std.Values[a];
            }

            f.Save(fnout);
        }


        void Run()
                {
            Console.WriteLine("Algorithm version: " + algorithmVersion);

                        // graph files
                        List<string> graphmls = new List<string>();
                        string graphmlDir = @"graphml";
                        foreach (var fn in Directory.GetFiles(graphmlDir))
                        {
                                if (fn.EndsWith("scale500.graphml"))
                                        graphmls.Add(fn);
                        }
                        // process in four parts
                        int partCount = 4;
                        for (int i = 0; i < partCount; ++i)
                                Process(graphmls, graphmls.Count * i / partCount,
graphmls.Count * (i + 1) /
partCount);

            foreach (var kv in arcData)
            {
                ArcData ad = kv.Value;
                Dictionary<int, int> c = new Dictionary<int, int>();
                c[-1] = ad.Directions.Count(_ => _ == -1);
                c[0] = ad.Directions.Count(_ => _ == 0);
                c[1] = ad.Directions.Count(_ => _ == 1);
                int verdict = GetVerdict(c[-1], c[0], c[1]);
                ad.Direction = verdict;
                ad.MajoritySize = c[verdict];
            }

            foreach (var fn in graphmls)
                CreateDirectedVersion(fn, arcData);

            string outGraphml = "whole_graph"+ versionStr +".graphml";
            string outGraphViz = "whole_graph" + versionStr + ".dot";
            CustomGraph outGraph = new CustomGraph();
            Dictionary<string, Node> nodeByName = new Dictionary<string, Node>();
            using (StreamWriter swGraph = new StreamWriter(outGraphViz))
            {
                swGraph.WriteLine("digraph directed_brain_graph {");
                using (StreamWriter sw = new
StreamWriter("all_arcs"+versionStr+".csv"))
                {
                    foreach (KeyValuePair<string, string> kv in rowForEdge)
                    {
```

```
                        string[] cells = kv.Value.Split(';');
                        int undefCount = 0, forwardCount = 0, backwardCount = 0;
                        for (int i = 0; i < partCount; ++i)
                        {
                            int cellindex = (i + 1) * 6 - 1;
                            if (cellindex >= cells.Length)
                                break;
                            int direction = int.Parse(cells[cellindex]);
                            switch (direction)
                            {
                                case -1: ++undefCount; break;
                                case 0: ++forwardCount; break;
                                case 1: ++backwardCount; break;
                            }
                        }
                        // directed edges: at least two cells in the same
direction,
                        // and at most 1 other cell
                        int verdict = GetVerdict(undefCount, forwardCount,
backwardCount);

                        sw.WriteLine(verdict + ";" + kv.Key + ";" + kv.Value);

                        string[] nodes = kv.Key.Split('~');
                        swGraph.WriteLine("edge[" + (verdict == -1 ? "dir = none, "
: "") + "color=red];");
                        if (!nodeByName.ContainsKey(nodes[0]))
                            nodeByName[nodes[0]] = outGraph.AddNode();
                        if (!nodeByName.ContainsKey(nodes[1]))
                            nodeByName[nodes[1]] = outGraph.AddNode();
                        switch (verdict)
                        {
                            case -1:
                                swGraph.WriteLine(nodes[0] + " -> " + nodes[1] +
";");
                                outGraph.AddArc(nodeByName[nodes[0]],
nodeByName[nodes[1]],
Directedness.Undirected);
                                break;
                            default:
                                swGraph.WriteLine(nodes[verdict] + " -> " +
nodes[1- verdict] + ";");
                                outGraph.AddArc(nodeByName[nodes[verdict]],
nodeByName[nodes[1- verdict]],
Directedness.Directed);
                                break;
                        }
                    }
                }
                swGraph.WriteLine("}");
            }

            // output the directed graph as a graphml
            GraphMLFormat graphmlOut = new GraphMLFormat();
            graphmlOut.Graph = outGraph;
            var irányított_nodeName = new StandardProperty<string>();
            irányított_nodeName.Domain = PropertyDomain.Node;
            irányított_nodeName.Name = "dn_name";
            foreach (var kv in nodeByName)
                irányított_nodeName.Values[kv.Value] = kv.Key;
            graphmlOut.Properties.Add(irányított_nodeName);
            for (int i = 0; i < 3; ++i)
            {
                var irányított_nodePos = new StandardProperty<double>();
                irányított_nodePos.Domain = PropertyDomain.Node;
                irányított_nodePos.Name = "dn_position_" + "xyz"[i];
                foreach (var kv in nodePosByName)
                    irányított_nodePos.Values[nodeByName[kv.Key]] = kv.Value[i];
                graphmlOut.Properties.Add(irányított_nodePos);
            }
            graphmlOut.Save(outGraphml);
        }
```

```
		static void Main(string[] args)
		{
			new Program().Run();
		}
	}
}
```
